# Deregulation of CSMD1 targeted by microRNA-10b drives gastric cancer progression through the NF-κB pathway

**DOI:** 10.7150/ijbs.23802

**Published:** 2019-08-06

**Authors:** Xiang-Liu Chen, Lian-Lian Hong, Kai-Lai Wang, Xiang Liu, Jiu-Li Wang, Lan Lei, Zhi-Yuan Xu, Xiang-Dong Cheng, Zhi-Qiang Ling

**Affiliations:** 1 Department of Digestive Oncology, the First Affiliated Hospital of Wenzhou Medical University; the First Provincial Wenzhou Hospital of Zhejiang, Wenzhou 325000.; 2 Zhejiang Cancer Institute, Institute of Cancer Research and Basic Medical Sciences of Chinese Academy of Sciences, Cancer Hospital of University of Chinese Academy of Sciences, Zhejiang Cancer Hospital, Hangzhou 310022, China.; 3 Department of Digestive Oncology, Zhejiang Province Cancer Hospital, Zhejiang Cancer Center, Hangzhou 310022, China.

**Keywords:** microRNA-10b, CUB and sushi multiple domains protein 1 (CSMD1), gastric carcinoma (GC), epithelial-mesenchymal transition (EMT), Tumor metastasis.

## Abstract

**Aim:** This study aimed to investigate the oncogenic activity of microRNA-10b by targeting CUB and sushi multiple domains protein 1 (CSMD1) in human gastric cancer (GC) and the underlying mechanisms.

**Methods:** The expression of CSMD1 in human GC tissues was evaluated by real-time reverse transcription polymerase chain reaction (RT-PCR), immunoblotting, and immunohistochemical analysis. The expressive abundance of microRNA-10b was detected by stem-loop RT-PCR. Molecular and cellular techniques, including lentiviral vector-mediated knockdown or overexpression, were used to elucidate the effect of microRNA-10b on the expression of CSMD1.

**Results:** CSMD1 was targeted and downregulated by microRNA-10b in human GC tissues and cells, and the down-regulated expression of CSMD1 contributed to poor survival. The knockdown of microRNA-10b expression inhibited cell proliferation in GC cells *in vitro* and tumor growth *in vivo.* The inhibition of microRNA-10b expression repressed invasion and migration of HGC27 cells and retarded GC cells metastasis to the liver in Balb/c nude mice. The up-regulated expression of microRNA-10b promoted the proliferation and metastasis of MKN74 cell *in vitro.* Intratumoral injection of microRNA-10b mimic also promoted the growth and metastasis of tumor xenografts in Balb/c nude mice. Mechanistically, microRNA-10b promoted the invasion and metastasis of human GC cells through inhibiting the expression of CSMD1, leading to the activation of the nuclear factor-κB (NF-κB) pathway that links inflammation to carcinogenesis, subsequently resulting in the upregulation of c-Myc, cyclin D1 (CCND1), and epithelial-mesenchymal transition (EMT) markers.

**Conclusions:** The findings established that microRNA-10b is an oncomiR that drives metastasis. Moreover, a set of critical tumor suppressor mechanisms was defined that microRNA-10b overcame to drive human GC progression.

## Introduction

Gastric cancer (GC) is the fifth most common tumor and the third deadliest from cancer worldwide, making up 7% of cases and 9% of deaths 1. It is particularly prevalent in East Asia, such as Japan, Korea, China, and so on 2. Despite a steadily declining incidence, it newly occurred in 950,000 people and caused 723,000 deaths in 2012 1. In the past few decades, although the diagnosis and treatment of gastric cancer has indeed improved significantly, the 5-year survival rate of patients with advanced gastric cancer, especially metastatic gastric cancer, is still very low 3. Hunman CUB and sushi multiple domains protein 1 (CSMD1) is a novel candidate tumor suppressor gene located on the p arm of chromosome 8 (8p23) 4. Previous studies have shown that the deletion of 8p23.2 or reduced expression of CSMD1 has something to do with poor prognosis in many cancers 5-9. Multiple mechanisms can reduce the expression of CSMD1. The allelic loss, copy number aberrations, mutations, and methylations of CSMD1 have been detected in various malignant tumours such as head and neck tumor, colorectal cancers, liver cancer, and so on 5-16. Besides, it has been proved that the change of microRNA expression and dysfunction play an important role in tumorigenesis and metastasis by regulating target genes and pathways, subsequently resulting in the alteration of proliferation, differentiation, apoptosis, invasion and metastasis of tumor cells 17-19. Therefore, the molecular mechanism underlying the microRNAs-driven deregulation of CSMD1 contributing to GC metastases need to be urgently unraveled.

MicroRNA is a small single-stranded non-coding RNA, that is a post-transcriptional negative regulator that binds completely or partly to the complementary sites in the 3′-untranslated region (3′UTR) of target mRNAs 17-19. Accumulating evidence has shown that miRNAs can modulate tumor growth, metastasis, and progression by regulating multiple target genes 20-22. microRNA-10b has been found to be highly expressed in many malignant tumours and related to the progress of breast cancer 23, colorectal cancer 24, head and neck cancer 25, pancreatic adenocarcinoma 26, glioblastoma 27, nasopharyngeal cancer 28, and liver cancer 29. The pleiotropic nature of microRNA-10b was due to its suppression of multiple tumor suppressors, including ras homolog family member C (RhoC), urokinase plasminogen activator receptor (uPAR), matrix metalloproteinases (MMPs), tumor protein p53 (TP53), forkhead box O3 (FOXO3), CYLD lysine 63 deubiquitinase (CYLD), paired box 6 (PAX6), patched 1 (PTCH1), homeobox D10 (HOXD10), notch receptor 1 (NOTCH1), BCL6 transcription repressor (Bcl-6), and Kruppel like factor 4 (KLF4) 29-33. A recent study reported that microRNA-10b mediated transforming growth factor-β1-regulated glioblastoma proliferation, migration, and epithelial-mesenchymal transition (EMT) 34. Moreover, the up-regulation of microRNA-10b is closely related to the survival of patients and is a predictors of poor prognosis in some cancer patients 35-37. This suggested that microRNA-10b could play a critical role in many types of human cancers.

Whether microRNA-10b acts as oncogene or tumor suppressor in GC is still not known. Kim et al. 38 reported that the expression of microRNA-10b was frequently silenced in GCs by its promoter methylation, and also microRNA-10b might act as a tumor suppressor by suppressing oncogenic microtubule associated protein RP/EB family member 1 (MAPRE1) at the transcriptional level, which was further confirmed by Li et al 39. However, Liu et al. reported that microRNA-10b stimulates the up-regulated expression of RhoC and AKT serine / threonine kinase (AKT) phosphorylation by targeting HOXD10, thus promoting the invasion of gastric tumors 40. Wang et al. also found that the expression of microRNA-10b was significantly up-regulated in GCs, which was related to tumor size, Lauren classification, invasive depth, lymphatic metastasis, distant metastasis, tumor- lymph node-metastasis (TNM) stage, and poor prognosis 41. In this study, we found that the expression of microRNA-10b was up-regulated in gastric cancer, which was positively correlated with lymph node metastasis. The overexpression of miR-10b promoted the proliferation and invasion of GC cells *in vitro* and the growth and liver metastasis of GC cells in nude mice. On the contrary, knockdown of microRNA-10b expression significantly inhibited the proliferation and invasion *in vitro*, and tumor growth of GC cells *in vivo.* The study concluded that miR-10b played an oncogenic role in GC metastasis.

## Material and methods

### Cell lines, clinical tissue specimens of GC

Six human GC cell lines (MKN74, AGS, BGC823, MKN28, SGC7901, HGC27) and a human fetal gastric mucosa epithelium cell line (GES-1) were purchased from the Cell Bank of the Chinese Academy of Sciences (Shanghai, P. R. China). Cells were grown in the Roswell Park Memorial Institute (RPMI)-1640 medium containing 10% fetal heat-inactivated bovine serum (FBS) 100 U/mL penicillin and 100 U/mL streptomycin (Gibco, Invitrogen Inc., CA, USA), maintained at 37°C incubator in a humidified atmosphere consisting of 5% CO_2_.

A total of 62 GC patients [mean age, 55.5 years; range, 38-72 years; 36 (58.1%) male and 26 (41.9%) female] who underwent radical resection of GC, without any preoperative treatment, at Institute of cancer research and basic medical sciences of Chinese Academy of Sciences, Cancer Hospital of University of Chinese Academy of Sciences, Zhejiang Cancer Hospital (No.1 Banshan East Road, Gongshu District, Hangzhou 310022, P. R. China.) from January 2011 to December 2012 were recruited in present study. All patients underwent pathological diagnosis by two professional pathologists independently according to the criteria of the American Joint Committee on Cancer (AJCC) / Union for International Cancer Control (UICC) TNM classification. Moreover, in 18 (29.0%), 31 (50.0%), and 13 (21.0%) patients, the tumors were found in gastric antrum, gastric body, and gastric cardia, respectively. Further, 14 (22.6%) patients had stage Ⅰ/Ⅱ GC and 48 (77.4%) had stage Ⅲ/Ⅳ GC. Based on the histological findings, 47 (75.8%) patients had an intestinal type and 15 (24.2%) had a gastric type cancer. The carcinoma was highly to moderately differentiated in 13 (21.0%) patients and poorly differentiated in 49 (79.0%) patients. None of the patients had any history of other tumors. Tumors and their paired paracancerous normal tissues were collected during surgery. The tissue specimens of this study were obtained with informed consent and were studied in accordance with the guidelines approved by Zhejiang Cancer Hospital Medical Ethics Committee (Hangzhou, China). The regular follow-up of all enrolled GC patients was performed by the special person until the end date of the project.

### Real-time reverse transcription polymerase chain reaction (RT-PCR)

One-step total RNA extraction TRIzol reagent (Invitrogen, Carlsbad, California, USA) was used for the extraction of total cellular RNA from the cultured cells or frozen tissues. Reverse transcription of mRNA was performed using the PrimeScript RT reagent Kit (Takara, Otsu, Japan) as described by the manufacture. For reverse transcription (RT) reactions, stem-loop primers were used for cDNA synthesis in microRNA-10b experiments. Then the quantitative polymerase chain reaction (PCR) was carried out with primers for the expression abundance detection of microRNA-10b, CSMD1, E-cadherin, Twist, Snail, Zeb1, Vimentin, NFκB-p65, NFκB-p50, nitric oxide synthase 2 (iNOS), CCND1, c-MYC, glyceraldehyde-3- phosphate dehydrogenase (GAPDH), and U6. Quantitative RT-PCR reaction in ABI 7500 fast RT-PCR system (ABI, CA, USA) was carried out using SYBR Green PCR Master Mix (Invitrogen, Carlsbad, California, USA), according to the standard quantitative PCR program. The primer sequences used are listed in Table S1. The conditions of PCR amplification were as follows: 95°C 15 minutes, then 40 cycles of 94°C 15 seconds, 60°C 30 seconds, and 70°C 35 seconds. The comparative cyclic threshold method was used to calculate the relative expression and normalize it to GAPDH or U6 small nuclear (sn) RNA. All experiments were performed in triplicate, and the median values were taken.

### Immunohistochemical analysis

Immunohistochemistry was performed as described in a previous study 42. After deparaffinization, the antigen was recovered in 0.01 M citric acid buffer, and then the activity of endogenous peroxidase was inactivated in 3% H_2_O_2_ in methanol for 10 mins. Nonspecific binding was blocked by incubation with 10% normal goat serum in phosphate-buffered saline (PBS) for 1 h at room temperature. Prior to the addition of an primary antibody, 10% of normal goat serum was incubated at room temperature in phosphate buffer (PBS) for 1 h to block non-specific binding reactions. The slides were incubated with primary antibodies against CSMD1 (Santa Cruz Biotechnology, Inc.2145 Delaware Ave Santa Cruz, CA. 95060, USA) overnight at 4°C, and then added biotinylated goat anti-mouse immunoglobulin G (Sigma, MO, USA) for 1 hour at room temperature. Then, a streptavidin-biotin-peroxidase complex assay was performed. The peroxidase activity was developed by incubating with 0.1% 3,3-diaminobenzidine (Sigma) in PBS with 0.05% H_2_O_2_ for 5 min at room temperature. As described methods in our previous studies 42, 43, based on the frequency and intensity of staining, the scores of histochemical staining were determined by three independent clinical pathologists, and the inconsistent results were finally determined after discussion.

### Western blotting analysis

The western blotting analysis was carried out using Immobilon-P polyvinylidene difluoride membranes (Millipore, 28820 Single Oak Drive, Temecula, California 92590, USA) as described in previous studies 42,43. The cell lysates were separated on sodium dodecyl sulfate-polyacrylamide gels and the western blotting analysis was carried out with following antibodies against CSMD1, Cyclin D1 (Santa Cruz Biotechnology), Snail (Abgent Inc, CA, USA), E-cadherin, Vimentin, Twist, Zeb1 (Cell Signaling Technology, MA, USA), iNOS, NFκB-p65, NFκB-p50 (Abcam, Cambridge, CB4 0FL, UK), and c-MYC (Sigma Chemical Co., MO, USA), respectively. β-actin (TaKaRa Co. Ltd, Kusachi, Japan) was used as an internal control. The density of band was measured using the ChemiDoc™ XRS^+^ System (1000 Alfred Nobel Drive, Hercules, California 94547, USA) equipping with Image-Pro Plus software and Epson color image scanner. The data was normalized to the β-actin.

### Plasmid construction

In order to study the overexpression of microRNA-10b, the primer pairs were designed to amplify a genome fragment of human microRNA-10b precursor using PCR method as described in a previous study 29. The PCR product was cloned into pcDNA3.1 (Gibco, Invitrogen Inc., CA, USA) and named as pcDNA3.1-microRNA-10b. The negative control of Hsa-microRNA-10b inhibitor and inhibitor came from GenePharma Company (Shanghai, China). The pmiR-RB-REPORT luciferase vector (GenePharma, Shanghai, China) was used to construct the pMIRCSMD1-3′-UTP - wild-type (WT) or pMIR-CSMD1-3′-UTP-mut vectors. WT and mutant inserts were confirmed by sequencing. The pSilencer-nuclear factor κB (NFκB)-p65-shRNA recombinant plasmid vector, containing short hairpin RNA (shRNA) interfering sequence aiming at the target of NFκB-p65 gene, was constructed. The NFκB-p65-shRNA sequence was constructed as follows: 5′-GATCCgccctatccctttacgtca*TTCAAGAGA*TGACGTAAAGGGATAGGGCttttttggaaa-3′; the lowercase part represents an interference sequence and the italic part represents a stem ring structure with the viscous ends of restriction endonuclease *Bam*HI and *Hind*Ⅲ at both ends. The constructed recombinant vector pSilencer-NFκB-p65-shRNA was sequenced, and the identified recombinant vector and negative control pSilencer were transfected into human GC cell line HGC27 by liposome transfection to verify its interference effect on NFκB-p65 gene, respectively. In the subsequent experiments, pSilencer-Negative Control (NC)-shRNA transfection was used as a negative control. The experiment was divided into three groups: pSilencer-NFκB-p65-shRNA transfection group, pSilencer-NC-shRNA transfection group (negative control group), and blank control group (only adding transfection reagent). The experiment was repeated three times.

### Cell transfection

The cultured cells of 1 × 10^5^ were plated to 50% confluence and transfected with the expression vector of microRNA-10b or the inhibitor lentiviral of hsa-microRNA-10b by Lipofectamine 2000 (Gibco, Invitrogen Inc., CA, USA) as described in previous studies 42. The inhibitor of microRNA-10b was chemically enhanced with 2′-*O*-4′-*C*-methylene modification. The expression vector of microRNA-10b and the inhibitors of microRNA-10b were designed and synthesized by a professional biology co., Ltd (GenePharma Inc. Shanghai, China). The sequence thus obtained was as described in a previous study 44. The transfection efficiency was detected by fluorescence image 24 hours later, and all kinds of experiments were carried out 48 hours later, and all the experiments were done in triplicate.

### Luciferase reporter assay

The firefly luciferase reporter gene constructs and miR-10b-expressing plasmid were transiently transfected into cells with 80% confluence in 24-well plates. After 48 hours, the luciferase activity was determined by Dual-Luciferase Reporter Assay kit (Promega, Madison, WI, USA) as described in Kit's instructions, and the luciferase activity was normalized to Renilla luciferase activity.

### Cell growth assay

For the 3-(4,5-Dimethylthiazol-2-yl)-2,5-diphenyltetrazolium bromide (MTT) assay, 1000 cells were inoculated in 96 well plate and transfected. The absorbance at 490 nm was measured once a day for 7 days. The soft agar assay is as follows: about 3000 cells were seeded in a 6-well plate and transfected. The size and number of soft agar colonies were detected after 14 days of transfection. All experiments were performed in triplicate, and the median values were taken.

### Migration assays

The cells (5 × 10^4^) were seeded into chambers with 8-μm pores (Corning Inc., NY, USA). The cells migrated to the bottom chamber through the membrane were fixed and stained with crystal violet after 24 hours. The numbers of migrating cells were counted by randomly selecting nine fields of view.

### Invasion assays

The cells were seeded in the 24-well BD BioCoat Matrigel Invasion Chamber (BD Biosciences Discovery Labware, MA, USA) at a density of 1 × 10^6^ per well. The serum (10%) was used as a chemoattractant. The membrane of the upper chamber was fixed and stained using a Diff-Quik reagent (Sysmex, Kobe, Japan) after 22 hours of incubation. The invaded cells were counted in four randomly selected sites per membrane.

### Tumor cell xenograft assay in nude mice

Six-week-old female BALB/c nude mice (weighing 20-22 g; Zhejiang Laboratory Animal Center, Hangzhou, China) were used in this study. All animal experiments were carried out in accordance with the agreement approved by the Animal Care and Use Committee of our Institute. For tumor growth experiments, 5×10^6^ cells were subcutaneously injected the lower back regions of 6-week-old female nude mice for 4 consecutive weeks, and 3 nude mice in each group. The tumor growth was measured by caliper every other day, and the tumor volume was calculated using the formula as: volume = length × width^2^ × 0.5. Finally, the mice were sacrificed and tumor mass was harvested for examination.

### Statistical analysis

All data were represented as mean ± standard deviation (SD) of at least three independent experiments. The difference among groups was analyzed using the paired *t* test for normal distribution by the *F* test. All statistical analyses were performed using SPSS 19.0 (SPSS Inc., IL, USA). A *P* value <0.05 was considered statistically significant. The survival curves and univariate analysis were carried out by the Kaplan-Meier method and log-rank test.

## Results

### CSMD1 was downregulated in GCs, which was related to patients' prognosis

**Figure 1:**
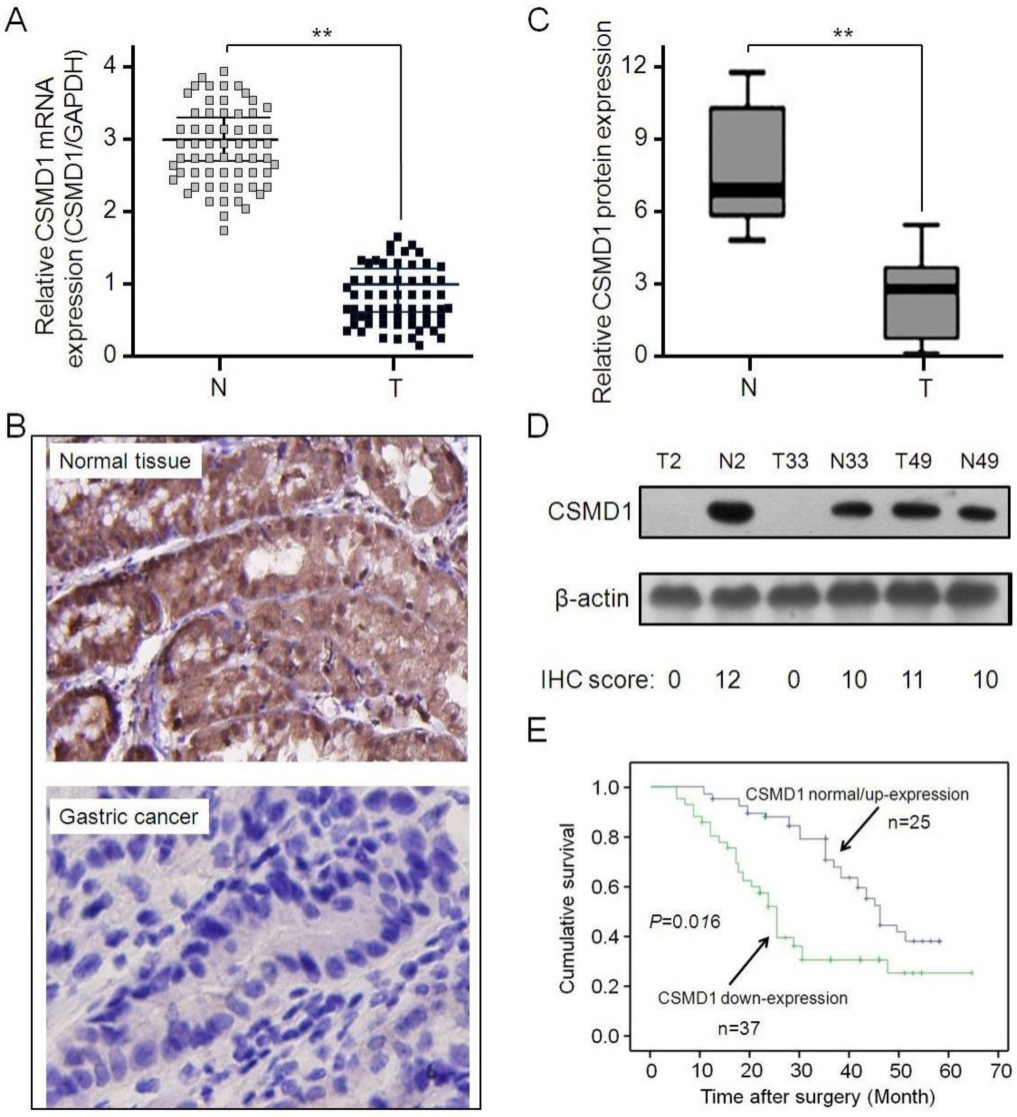
** The expression of CSMD1 is downregulated in gastric tumor tissues and related to the survival of patients. (A)** The relative levels of CSMD1 in sixty-two paired of GC samples were measured by real-time quantitative RT-PCR, and the GAPDH was used as an internal control. Student's t test was used to analyze the significant differences between the GC and normal tissues, **P < 0.01. **(B)** The expression levels of CSMD1 were detected by immunochemistry analysis in 62 pairs of gastric tumor and matched adjacent normal tissues. Representative photos were shown in paired normal and tumor tissues (×200). **(C)** The comparison of staining scores of CSMD1 between gastric tumor and adjacent tumor free tissues, **P < 0.01. **(D)** Western blot analysis of CSMD1 protein in GC and paired normal tissues. Representative photos were shown in 3 paired tumor (T) and normal (N) tissues. The comparison of CSMD1 protein expression between IHC staining scores and western blot results. **(E)** Survival curves were plotted based on the Kaplan-Meier survival analysis. The expression level of CSMD1 was used as the variate to separate two lines (*P* = 0.016).

The expressive abundance of CSMD1 was determined in patients with GC by RT-PCR. CSMD1 was significantly downregulated in GC tissues compared with normal tissues (Figure 1A). This result was supported by the immunohistochemical staining (Figure 1B and 1C) and Western blot analysis (Figure 1D). Subsequently, the relationship between the expression of CSMD1 in GC tissues and their overall survival was investigated. The lower expression of CSMD1 in GC tissues was associated with the shorter survival time during 5-year follow-up (*P* = 0.016, Kaplan-Meier survival and log-rank test) (Figure 1E), suggesting that the level of CSMD 1 in GC tissues was negatively correlated with patient's survival.

### Expressive abundance of microRNA-10b in GC tissues and cell lines

**Figure 2:**
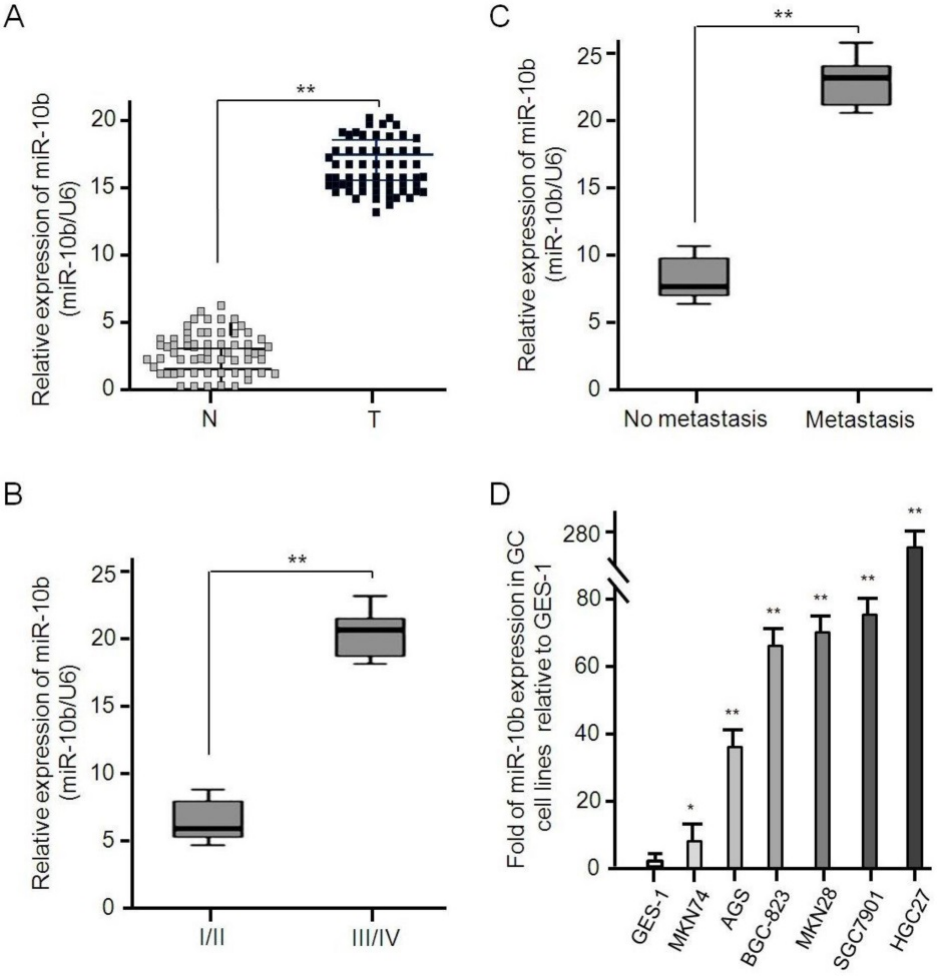
** miR-10b is up-regulated in human GC tissues and cell lines. (A)** The expression of miR-10b in tumor and matched normal tissues from 62 GC patients was analyzed by real-time PCR, **P < 0.01. **(B)** The miR-10b expression was significantly higher in patients with stage III/IV than in those with stage I/II, **P < 0.01. **(C)** The miR-10b expression was significantly higher in patients with metastasis than in those without metastasis, **P < 0.01. **(D)** The relative miR-10b expression normalized by U6 in seven GC cell lines was detected by real-time RT-PCR. Data were presented as fold change in GC cell lines relative to human normal gastric epithelial cell line GES-1 and the results from three independent experiments are shown in the right as mean ± SD. *P < 0.05, **P < 0.01.

The expressive abundance of microRNA-10b was determined in 62 pairs of GC tissues and its matched adjacent normal tissues, and U6 used to normalize the expression. As shown in Figure 2, the expression of microRNA-10b in GC tissues was significantly higher than that in adjacent normal tissues (mean ± SD: 17.524 ± 3.286 vs 3.307 ± 1.875, *P* < 0.001; Figure 2A). Considering TNM stage, the expressive abundance of microRNA-10b in GC tissues was positively correlated with advanced TNM stage (*P* < 0.001, Figure 2B). Moreover, the expression level of microRNA-10b in GC patients with metastasis, including lymph node metastasis, was significantly higher than that in those without metastasis (*P* < 0.001, Figure 2C). Similarly, the expression level of microRNA-10b in GC cell lines MKN74, AGS, BGC 823, MKN28, SGC7901 and HGC27 cells was significantly higher than that in human gastric epithelial cell line GES-1 cells (Figure 2D).

### Oncogenic role of microRNA-10b in GC cells

**Figure 3:**
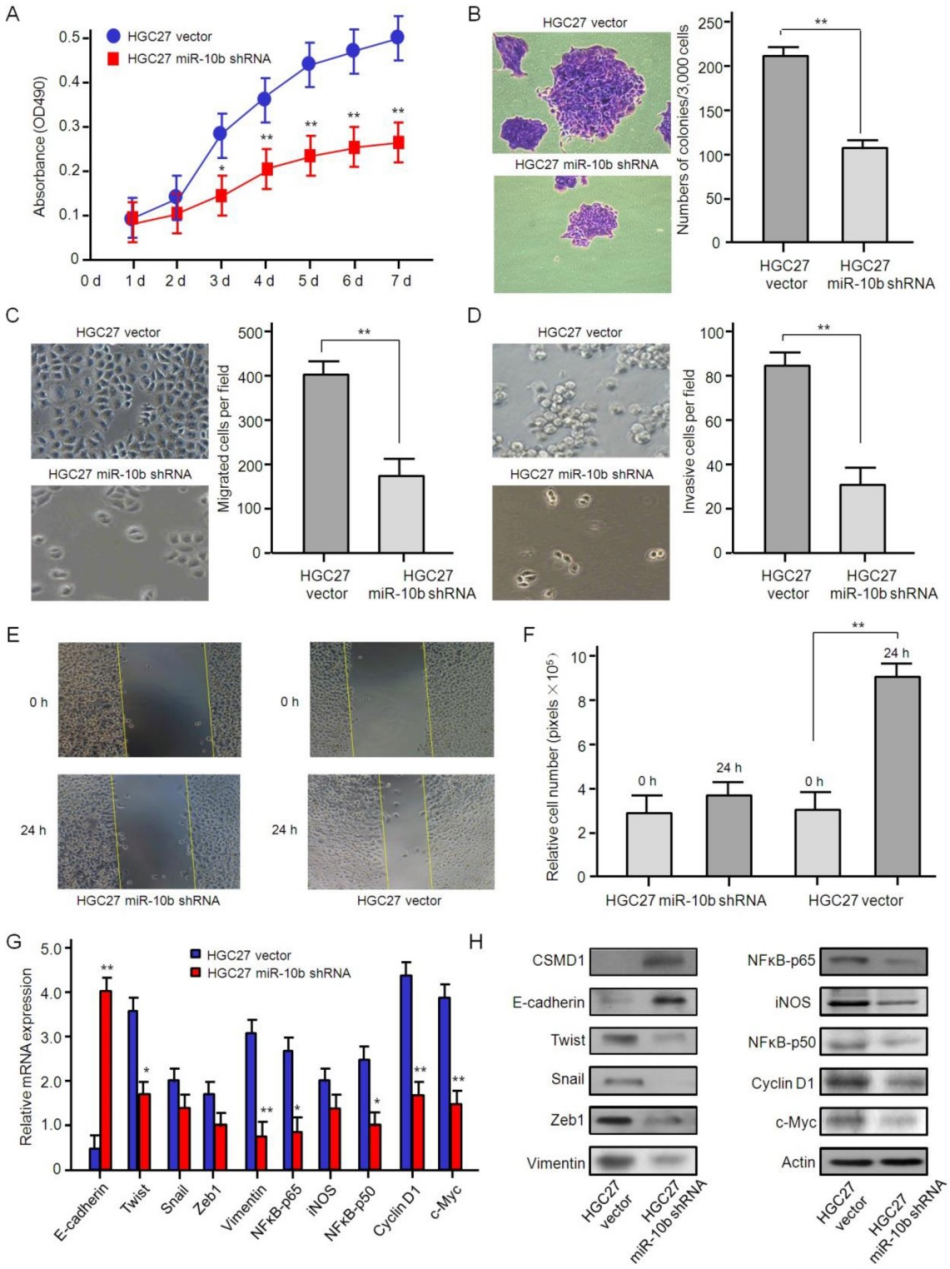
** The knockdown of miR-10b inhibited gastric cancer cell growth, migration, and invasion *in vitro.* (A)** The effect of miR-10b downexpression on cell proliferation in HGC27 was determined by the MTT assay. The data are calculated from triplicate experiments and shown as mean ± SD. **(B)** Representative images show the colony formation of HGC27 cells with miR-10b shRNA and their control cells (left panel). Average colonies in each well for each group were counted from three independent experiments and shown as mean ± SD (right panel). **(C, D, E, F)** The effects of miR-10b downexpression on cell migration, invasion and metastasis in HGC27 cells were analyzed by Transwell migration, Matrigel-coated Transwell invasion analyses and a wound healing assay. For Transwell assay and Matrigel-coated Transwell invasion analyses, the migrated cells on the bottom surface of each well were fixed and stained and the representative images are shown (×100). The area of migrated or invading cells per field was quantified and shown in the right as mean ± SD (n = 8). * and ** indicate *P* < 0.05 and *P* < 0.01 respectively between the corresponding groups in “HGC27 vector'' vs ''HGC27 miR-10b shRNA''. For wound healing assay, representative images of HGC27 cells at 24 h after wound scratching are shown (×200) and the results from three independent experiments are shown in the right as mean ± SD. **P < 0.01. **(G)** Real-time RT-PCR analysis of EMT- and NFκB pathway-related genes. The data are calculated from triplicate experiments and shown as mean ± SD. *P < 0.05, **P < 0.01. **(H)** Western blot analysis of EMT- and NFκB pathway-related genes with the antibodies as indicated.

In order to evaluate the biological function of microRNA-10b in GC cells, HGC27, a GC cell line with high expression of endogenous microRNA-10b, was stably transferred into microRNA-10b inhibitor by lentivirus infection. MTT analysis showed that the knockdown expression of microRNA-10b significantly inhibited the proliferation of HGC 27 cells* in vitro* (Figure 3A). Soft agar assay showed that the knockdown expression of microRNA-10b significantly inhibited the colony expansion of HGC 27 cells (Figure 3B). Then, the effects of microRNA-10b expression on migration ability of GC cells were studied by Transwell. The knockdown expression of microRNA-10b in HGC27 cells significantly repressed cell migration (Figure 3C). Also, the effect of microRNA-10b expression on the invasive properties of HGC27 cells was studied by Matrigel transmembrane invasion assay. The knockdown expression of microRNA-10b significantly reduced the invasiveness of HGC27 cells (Figure 3D). In wound healing experiment, HGC27 cells treated with miR-10b shRNA significantly reduced cell migration (Figure 3E and 3F). In order to investigate the potential mechanism associated with these phenotypic changes, the expressions of E-cadherin, Twist, Snail, Zeb1, Vimentin, NFκB-p65, iNOS, NFκB-p50, Cyclin D1, and c-Myc in these samples at the mRNA and protein levels were further detected. The results showed that the mRNA and protein levels of Twist, Snail, Zeb1, Vimentin, NFκB-p65, iNOS, NFκB-p50, Cyclin D1, and c-Myc were downregulated, while the expression level of E-cadherin was increased, with the inhibition of microRNA-10b expression (Figure 3G and 3H). It was suggested that microRNA-10b promotes the ability of proliferation, migration and invasion of GC cells by regulating the EMT process, and then promotes the growth and metastasis of GC.

**Figure 4:**
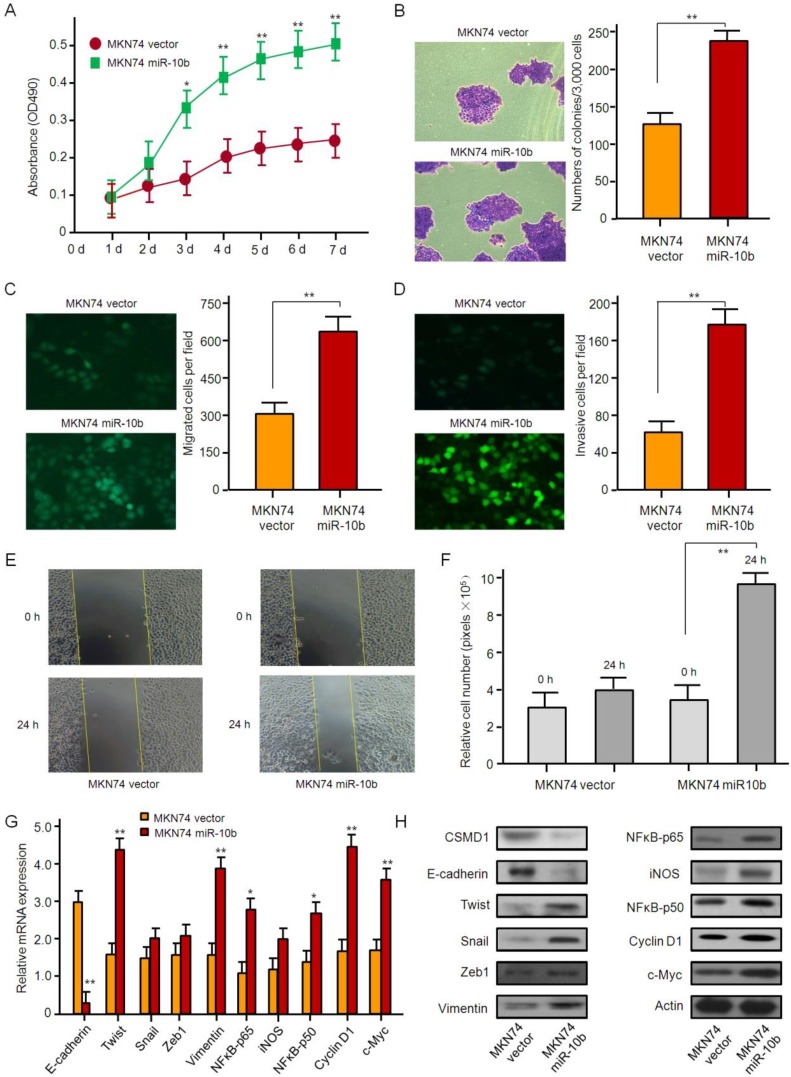
** The overexpression of miR-10b promotes gastric cancer cell growth, migration, and invasion *in vitro.* (A)** The effect of miR-10b overexpression on cell proliferation in MKN74 cells was determined by the MTT assay. The data are calculated from triplicate experiments and shown as mean ± SD. **(B)** Representative images show the colony formation of MKN74 cells with miR-10b overexpression and their control cells (left panel). Average colonies in each well for each group were counted from three independent experiments and shown as mean ± SD (right panel). **(C, D, E, F)** The effects of miR-10b overexpression on cell migration, invasion and metastasis in MKN74 cells were analyzed by Transwell migration, Matrigel-coated Transwell invasion analyses, with immunofluorescence staining, and a wound healing assay. For Transwell assay and Matrigel-coated Transwell invasion analyses, the migrated cells on the bottom surface of each well were fixed and stained and the representative images are shown (×100). The area of migrated or invading cells per field was quantified and shown in the right as mean ± SD (n = 8). * and ** indicate *P* < 0.05 and *P* < 0.01 respectively between the corresponding groups in “MKN74 vector'' vs ''MKN74 miR-10b''. For wound healing assay, representative images of HGC27 cells at 24 h after wound scratching are shown (×200) and the results from three independent experiments are shown in the right as mean ± SD. **P < 0.01. **(G)** Real-time RT-PCR analysis of EMT- and NFκB pathway-related genes. The data are calculated from triplicate experiments and shown as mean ± SD. *P < 0.05, **P < 0.01. **(H)** Western blot analysis of EMT- and NFκB pathway-related genes with the antibodies as indicated.

The ability of proliferation, invasion, and metastasis of MKN74 cells, whose endogenous microRNA-10b was normally expressed, was strengthened by microRNA-10b overexpression with lentiviral infection. The results of the MTT and soft agar assay showed that the overexpression of microRNA-10b promoted the proliferation and the colony formation of MKN74 cells (Figure 4A and 4B). Similar data were obtained by Transwell analysis. The overexpression of microRNA-10b promoted invasion and migration of MKN74 cells (Figure 4C-4F). The overexpression of microRNA-10b enhanced the ability of migration and invasion of MKN74 cells (Fig. 4C-4F), up-regulated the expression of Twist, Snail, Zeb1,Vimentin, NFκB-p65, iNOS, NFκB-p50, Cyclin D1, and c-Myc at the mRNA and protein levels, while the expression of E-cadherin was significantly down-regulated (Figure 4G and 4H).

In order to investigate the effect of down-regulation of NF-κB p65 on the cell proliferation and invasion of GC cells, a recombinant plasmid vector containing shRNA interference sequence and targeting NF-κB p65 gene was constructed and transformed into HGC 27 cells. The results showed that there was no significant difference in the proliferation ability between the pSilencer-NF-κB p65-shRNA transfected group and the pSilencer-Nc shRNA transfected group and the blank control group 12 h after transfection (both *P* > 0. 05). 24 h after transfection, the proliferation ability of the pSilencer-NF-κB p65-shRNA transfected group was significantly lower than that of the pSilencer-NC-shRNA transfected group and the blank control group (both *P* < 0.05). There was no significant difference in proliferation ability between the pSilencer-NC-shRNA transfected group and the blank control group (*P* > 0.05). The results showed that the proliferation activity of HGC27 cells was inhibited after decreasing the expression of NF-κB p65, that is, NF-κB p65 could promote the proliferation of GC cells. The results of the Transwell chamber invasion assay demonstrated that the number of perforated cells in the pSilencer-NF-κB p65-shRNA transfection group was 35.8 ±5.4. Compared with the pSilencer-NC-shRNA transfection group (68.2 ±7.6) and blank control group (65.6 ±7.2), the difference was statistically significant (both *P* < 0. 05). But, there was no significant difference in the number of transmembrane cells between the pSilencer-NC-shRNA transfection group and the blank control group (*P* > 0.05). These results indicated that NF-κB p65 could promote the invasion ability of HGC27 cells.

### microRNA-10b directly targeted CSMD1 in GC cells

**Figure 5:**
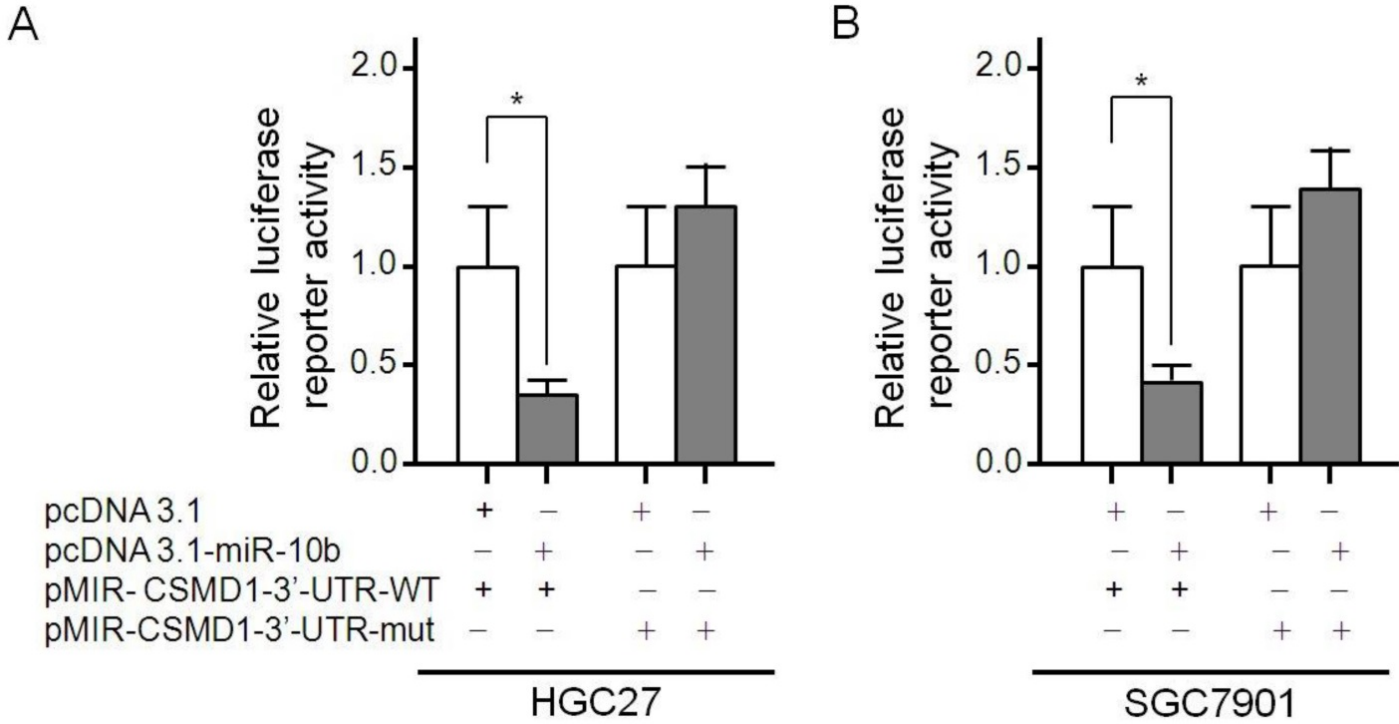
** The CSMD1-3′-UTR is a target of miR-10b. The diagram of the luciferase reporter plasmids:** plasmid with the full length wild-type CSMD1-3′-UTR (pMIR- CSMD1-3'-UTR-WT) insert and plasmid with a mutant CSMD1- 3′-UTR (pMIR-CSMD1-3'-UTR-mut) which carried a substitution of seven nucleotides (TGTCCCA) within the miR-10b binding site (707-713). Luciferase activity assay demonstrates a direct targeting of the 3′-UTR of CSMD1 by miR-10b. HGC27 and SGC7901 cells were transfected with pcDNA3.1-miR-10b and pMIR-CDMD1-3′- UTR-WT or pMIR-CSMD1-3′-UTR-mut. (**A**) HGC27 cells; (**B**) SGC7901 cells.

It has been reported that CSMD1 is regulated by microRNA-10b in human hepatocellular carcinoma cells 45. The Luciferase reporting assays were carried out to determine whether microRNA-10b was directly targeted CSMD1 in GC cells. The microRNA-10b binding site 707-713 was identified in the CSMD1 3′-UTR. pMIR-CSMD1-3′-UTR-WT contained the 707-713 binding sites. pMIR-CSMD1-3′-UTR-mut contained a mutation in the 707-713 (TGTCCCA) site. Therefore, a luciferase reporter assay was performed to confirm the binding ability of microRNA-10b to CSMD1 cDNA. It was found that the overexpression of microRNA-10b significantly inhibited the expression of luciferase in HGC27 cells transfected with pMIR-CSMD1-3′-UTR-WT but not with pMIR-CSMD1-3′-UTR-mut (Figure 5A). The luciferase activity of HGC 27 cells co-transfected with pMIR-CSMD1-3′-UTR-WT and pcDNA3.1-microRNA-10b was 0.38-fold lower than that of the negative control group (*P* < 0.05). A similar effect was also observed in SGC7901 cells (a 0.41-fold decrease compared with the blank control, *P* < 0.05; Figure 5B). These results suggest that microRNA-10b binds to the 707-713 site of CSMD 1 3'-UTR in GC cells.

### microRNA-10b promoted growth and metastasis of gastric cancer *in vivo*

**Figure 6:**
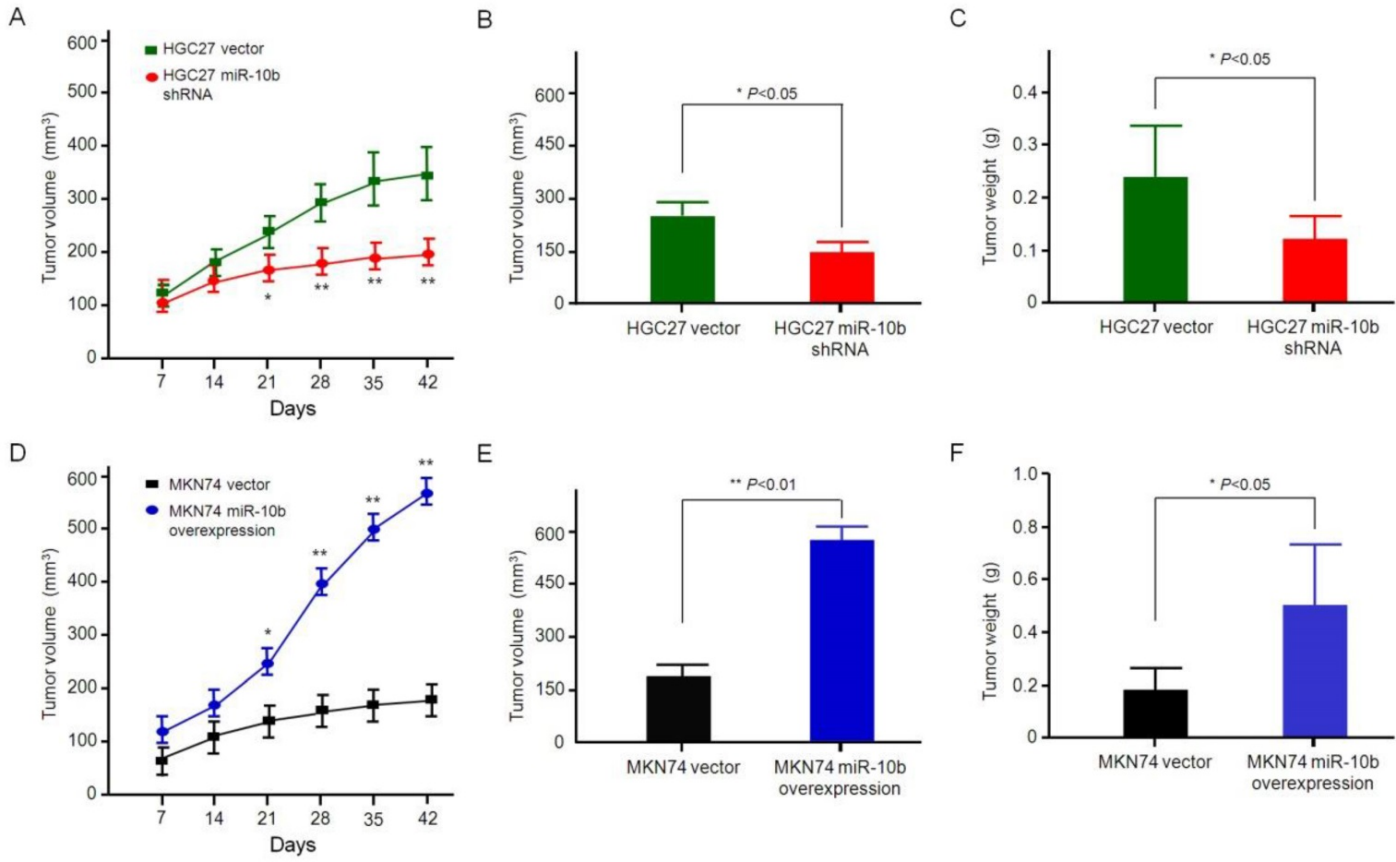
** Effect of miR-10b expression on GC growth *in vivo.*** (**A**,** D**) Determination of tumor volumes at different time points in (**A**) HGC27 miR-10b shRNA *vs* HGC27 vector, (**D**) MKN74 miR-10b overexpression* vs* MKN74 vector. The data are calculated from 3 nude mice per group and shown as mean ± SD. *P < 0.05, **P < 0.01. (**B**,** E**) After the final measure, the mice were sacrificed, and the tumors were excised. Tumor volume was measured and calculated using the formula length × width^2^/2. Student's *t* test was used to analyze the significant differences. The data are calculated from 3 nude mice per group and shown as mean ± SD. *P < 0.05, **P < 0.01. (**B**) HGC27 miR-10b shRNA *vs* HGC27 vector, (**E**) MKN74 miR-10b overexpression* vs* MKN74 vector. (**C**,** F**) After the final measure, the mice were sacrificed, and the tumors were excised. Tumor weight was measured and the Student's *t* test was used to analyze the significant differences. The data are calculated from 3 nude mice per group and shown as mean ± SD. *P < 0.05, **P < 0.01. (**C**) HGC27 miR-10b shRNA *vs* HGC27 vector, (**F**) MKN74 miR-10b overexpression* vs* MKN74 vector.

In order to determine whether microRNA-10b can promote the growth and metastasis of GC cells *in vivo,* two groups of GC models, such as HGC27 cells with microRNA-10b shRNA and control group cells, MKN74 cells with the overexpression of microRNA-10b and control group cells thereof, were established. Then, these cells were injected subcutaneously into the nude mice. After injection of a combination of HGC27 cells with microRNA-10b shRNA for 6 weeks, the tumor volume and weight of mice were smaller and lighter than those of the mice injected with HGC27 vector cells (Figure 6A, 6B and 6C). In addition, the mice in the control group showed obvious primary tumor, while the tumor volume and weight of the mice with combined injection of MKN74 cells and microRNA-10b overexpression was significantly increased (Figure 6D, 6E and 6F). By comparing the data of microRNA-10b overexpression group or microRNA-10b knockdown group and their control group at different experimental points, it was concluded that microRNA-10b might result in an average increase of the tumor growth. Only orthotopic tumors were found in the HGC27 miR-10b shRNA and MKN74 vector groups, whereas tumor metastasis was found in 66.7% (2/3) of the MKN74 miR-10b-overexpressed group and 33.3% (1/3) of the HGC27 vector group. These results suggested that miR-10b promoted the metastasis of GC cells.

## Discussion

Metastasis is a complex multistep process triggered by a body of transcriptive factors. microRNA plays an important role in regulating gene expression 20-22. Aberrant microRNA expression level in various human cancers contributes to tumor progression at different stages by inhibiting their target genes 46. Therefore, the recognition of specific microRNA and its targets related to tumorigenesis and metastasis can provide clues for tumor diagnosis, treatment and prevention. microRNA-10b is located in the HOX gene cluster on chromosome 2, which is closely related to tumor invasion and metastasis. Previous studies have shown that microRNA-10b is overexpressed in breast cancer 23, colorectal cancer 24, head and neck cancer 25, pancreatic cancer 26, glioblastoma 27, nasopharyngeal carcinoma 28, hepatocellular carcinoma 29 and so on. Consistent with previous reports, this study showed that the expression of microRNA-10b in GC tissues and 6 GC cell lines was higher than that in paracancerous non-tumor tissues and GES-1 cell line. Besides, the expression of microRNA-10b in patients with metastasis, including lymph node metastasis, was significantly higher than that in patients without metastasis. The up-regulation of microRNA-10b has been shown to promote the invasion and metastasis of various tumors 23-29. In this study, the knockdown of miR-10b expression inhibited the growth, migration and invasion of HGC27 cells. The converse was also true. Overexpression of microRNA-10b promoted the growth, migration and invasion of MKN74 cells. Taken together, these results were consistent with the previous findings, showing that microRNA-10b promoted the metastasis of GC 38-41, suggesting that microRNA-10b exerted oncogenic activity in GC cells, however, the mechanism by which it drives tumor metastasis is unclear. Previous studies have revealed that microRNA-10b promotes the metastasis of a variety of tumor cells by regulating Bim, TFAP2C, P16, P21, E-cadherin, CD138, RHOC, RhoC, uPAR, MMP-2, MMP-9, HOXD10, etc 23-29. In this study, microRNA-10b was involved in the proliferation and metastasis of GC cells by targeting CSMD1, and facilitated epithelial-mesenchyma transformation (EMT) process by the NF-κB pathway.

CSMD1 is localized on chromosome 8p23.2, which is a putative tumor suppressor gene 4. Mounting evidence indicates that the deletion of 8p23.2 or reduced expression of CSMD1 is associated with the development of many cancers 5-9. Multiple mechanisms can reduce the expression of CSMD1. The allelic loss, mutation and methylation of CSMD1 have been detected in breast cancer, head and neck cancer, oral squamous cell carcinoma, prostate cancer, rectum, liver, lung and skin cancer and many other malignant tumors such as breast cancer, head and neck cancer, oral squamous cell carcinoma, prostate, colorectal cancer, liver cancer, lung cancer and skin cancer, etc 5,6,8-16. Using four databases of TargetScan, Miranda, miRWalk and PicTar, highly conserved microRNA-10b binding sites were found in CSMD1 3′-UTRs. In our study, the molecular mechanism of the correlation between miR-10b and CSMD1 was further was further investigated. The results revealed that miR-10b could bind to the 707-713 binding sites in the 3′-UTRs of CSMD1. It was found that the expression of microRNA-10b in GC cells was negatively correlated with the expression of CSMD1, suggesting that microRNA-10b could promote the proliferation and metastasis of GC cells at least partly by down-regulating the expression of CSMD1.

In a word, the expression of microRNA-10b in GC tissues and cell lines were analyzed, and its effect on cells was studied. It was revealed that the overexpression of microRNA-10b enhanced the vitality, migration and invasion of GC cells. The study provided evidence that CSMD1 is indeed a direct target of microRNA-10b in GC cells, and microRNA-10b could mediate an oncogenic effect in GC by targeting CSMD1 expression. In addition, this study also provided potential diagnostic and prognostic markers and promising candidates for effective GC therapeutic strategies.

## Supplementary Material

Supplementary figures and tables.
