# Supplementary material for: Deregulation of CSMD1 targeted by microRNA-10b drives gastric cancer progression through the NF-κB pathway
Source: Int J Biol Sci. 2019 Aug 6;15(10):2075–86. doi: 10.7150/ijbs.23802 (PMC6775299; doi:10.7150/ijbs.23802)
Supplement: Supplementary file 1 — Supplementary figures and tables. [file ijbsv15p2075s1.pdf]

Supplementary Table 1. Primer sequences for real-time PCR

Genes	Forward primer (5'→3')	Reverse primer (5'→3')
miR-10b	cgt cgt acc ctg tag aac cga	gtg cag ggt ccg agg t
miR-10b-reverse	gtc gta tcc agt gca ggg tcc gag gta ttc gca ctg gat acg acc aca aa	
CSMD1	aga tgc tgc cgt caa aag atg gat	tca ctt tgt ctg ggt ccg ttg ttg
E-cadherin	cga gag cta cac gtt cac gg	ggg tgt cga ggg aaa aat agg
Twist	gcc tag agt tgc cga ctt atg	tgc gtt tcc tgt taa ggt agc atg
Snail	tcg gaa gcc taa cta cag cga	aga tga gca ttg gca gcg ag
Zeb1	tta cac ctt tgc ata cag aac cc	ttt acg att aca ccc aga ctg c
Vimentin	gcc cta gac gaa ctg ggt cag	ggc tgc aac tgc cta atg ag
NFκB-p65	gtg ggg act acg acc tga atg	ggg gca cga ttg tca aag atg
NF κB-p50	aac aga gag gat ttc gtt tcc g	ttt gac ctg agg gta aga ctt ct
iNOS	agg gac aag cct acc cct c	ctc atc tcc cgt cag ttg gt
Cyclin D1	ccg tcc atg cgg aag atc	atg gcc agc ggg aag ac
c-MYC	gca gct gct tac acg ctg ga	cgc agt aga aat acg gct gca c
GAPDH	ctg ggc tac act gag cac c	aag tgg tcg ttg agg gca atg
U6 snRNA	gct tcg gca gca cat ata cta aaa t	cgc ttc acg aat ttg cgt gtc at
U6-reverse	cgc ttc acg aat ttg cgt gtc at	
